# The Prediction of Body Mass Index from Negative Affectivity through Machine Learning: A Confirmatory Study

**DOI:** 10.3390/s21072361

**Published:** 2021-03-29

**Authors:** Giovanni Delnevo, Giacomo Mancini, Marco Roccetti, Paola Salomoni, Elena Trombini, Federica Andrei

**Affiliations:** 1 Department of Computer Science and Engineering, University of Bologna, 40127 Bologna, Italy; giovanni.delnevo2@unibo.it (G.D.); paola.salomoni@unibo.it (P.S.); 2 Department of Education, University of Bologna, 40127 Bologna, Italy; giacomo.mancini7@unibo.it; 3 Department of Psychology, University of Bologna, 40127 Bologna, Italy; elena.trombini@unibo.it

**Keywords:** obesity, depression, machine learning, artificial intelligence, reproducibility

## Abstract

This study investigates on the relationship between affect-related psychological variables and Body Mass Index (BMI). We have utilized a novel method based on machine learning (ML) algorithms that forecast unobserved BMI values based on psychological variables, like depression, as predictors. We have employed various machine learning algorithms, including gradient boosting and random forest, with psychological variables relative to 221 subjects to predict both the BMI values and the BMI status (normal, overweight, and obese) of those subjects. We have found that the psychological variables in use allow one to predict both the BMI values (with a mean absolute error of 5.27–5.50) and the BMI status with an accuracy of over 80% (metric: F1-score). Further, our study has also confirmed the particular efficacy of psychological variables of negative type, such as depression for example, compared to positive ones, to achieve excellent predictive BMI values.

## 1. Introduction

Obesity constitutes a major public health concern globally, generating considerable direct and indirect costs, and affecting over one-third of the world’s population [1]. Obesity is recognized as a complex, multifactorial disease, determined by a combination of factors and impacting both physical and psychological health [2]. However, existing research generally treats this condition mainly as a result of both behavioral factors, namely, an excessive caloric intake relative to metabolic energy expenditure [3], and genetic influences, such as single gene mutations [4]. The role of other relevant determinants, including psychological ones, tends therefore to be neglected although these variables clearly also contribute to weight gain and weight-related pathologies. Nevertheless, particularly in the field of psychology, researchers have emphasized a mutual association between overweight, obesity and high levels of negative affectivity, operationalized mainly as depression. For example, adults diagnosed with obesity report higher depression and anxiety levels compared to normally weighted individuals [5,6], and negative affects emerged as important factors for the maintenance of eating pathology [7]. At the same time, the literature shows inconsistencies with regards to strength and causal direction of such associations [8].

These mixed results might be related to several factors, including specific methodological issues, viz., the variables being measured, assessment tools, and strategies to data management. In fact, the tendency to employ a limited conceptualization of obesity, together with the general application of conventional regression analyses (e.g., linear and logistic regressions) to test empirical assumptions, reinforce existing difficulties in predicting and treating obesity. The use of regressions have certainly helped to identify risk factors of medical outcomes, however, in the case of a multidimensional, lifestyle condition such as obesity, these methods have made less progress [9].

One of the main approaches that may help to reduce these research-flaws and to improve scientific knowledge is the use of artificial intelligence (AI). In health-related disciplines, there is currently an increasing interest in the use of AI, particularly when the primary task is identifying clinically useful patterns in high-dimensional data sets. For example, several studies employed AI to classify a number of medical parameters that could efficiently predict obesity and body mass index (BMI; weight in kilograms divided by the square of the height in meters) [10], while a recent systematic review showed the application of machine learning (ML) algorithms for childhood obesity care [11].

Detection and diagnosis of diseases by the use of AI, in particular ML, is indeed an ongoing and prominent topic in scientific papers [12]. The interest in its potentiality has increased, even if the possible unintended consequences that may result from its application in clinical practice are clear, and include an overreliance on the capabilities of automation, thus reducing the skills of physician, as well as relying more on the data than on the clinical context [11]. Several medical investigations employed ML approaches to develop advanced remote healthcare systems to monitor long-term patients with BMI-related chronic illnesses [13,14,15,16]. Specifically, while a number of these studies attempted to predict BMI by voice signals [15], face images [16,17], or face points extracted with a Kinect [18], other studies focused on blood and biochemical indexes [19,20,21]. However, to our knowledge there are currently no studies analyzing the relationship between psychological functioning and BMI values through ML techniques.

Here, we aim to address this gap by further exploring the relationship between affect-related psychological variables and BMI through ML algorithms. Specifically, we applied ML to infer predictive features related to psychological functioning over BMI using data from a study [22] that demonstrated, employing correlational analysis, that depression levels may be useful in order to discriminate among BMI levels (normal weight, overweight, and all obesity classes). The main contributions of this work are twofold. Firstly, this study attempts to reproduce the results obtained on the relationship between affect-related psychological variables and BMI [22] by using ML techniques. Computational reproducibility is the ability to repeat an analysis of a given data set and obtain sufficiently similar results [23,24]. Not only is reproducibility critical for ML research [25], but it also constitutes a necessary requirement for science in general, given the constantly increasing need to subject study findings to more intensive scrutiny [26]. Secondly, this study aims to test whether psychological variables can be used as predictors to forecast unobserved BMI values [27]. The main objective of this study is therefore to identify risk and/or protective factors, conceptualized as negative and positive affectivity respectively, for overweight and obesity. Depending on the evidence for causality, these factors can be useful for screening patients who are at risk in a broader population as well as for the development of therapeutic interventions.

## 2. Materials and Methods

This section details the research questions at the base of this study, illustrates the dataset and the machine learning algorithms used, and then describes the employed approach together with the evaluation metrics adopted.

### 2.1. Research Questions

As anticipated in the Introduction section, the purpose of this study is to deepen the relationship between risk and protective factors, in the form of negative and positive affectivity for overweight and obese people through the use of Machine learning algorithms. In particular, the research questions that drove our study are the following ones:

(1) Is it possible to predict the BMI value (or the BMI class) using psychological variables?
(2) Which psychological variables, the positive or the negative ones, allow to better predict the BMI?
(3) Among them, which one has more influence on the prediction capability?

To answer these research questions, we followed the steps outlined below. Firstly, we used all the psychological variables as input to predict the BMI. Secondly, we considered separately the positive and the negative ones. This had let us to understand which ones allow to better predict the BMI. Finally, we evaluated those ones that work better following a leave-one-out approach to understand if one of them is more related than other ones to the BMI.

### 2.2. Dataset Description

The dataset used is composed of psychological variables exhibited by adults seeking treatment for their obesity, and by the control group. A detailed description of both participants and data collection procedure for this study is available in a recently published article [22]. The dataset comprised a set of both positive and negative psychological variables relative to 320 subjects. Positive variables were those psychological factors that may play a protective role against obesity and include trait emotional intelligence (trait EI) measured with the Trait Emotional Intelligence Questionnaire–Short Form [27]; cognitive reappraisal as emotion regulation strategy measured with the Emotion Regulation Questionnaire [28]; and happiness, measured with the Oxford Happiness Inventory [29]. Negative variables were instead potential risk-factors for the development and maintenance of obesity, and included: expressive suppression as emotion regulation strategy, binge eating, assessed with the Binge Eating Scale [30], depression, assessed with the Beck Depression Inventory [31], trait and state anxiety, assessed with the State Trait Anxiety Inventory-Y [32]. Each of these questionnaires used to measure a certain psychological variable returns an integer value. Hence, for each subject, there are seven different values representing the psychological state of the subject. In addition to these ones, there are the BMI obtained for each participant and BMI categories computed according to the BMI ranges given by the World Health Organization [33]. The subjects were organized into three groups: normal weight, overweight, and obese adults (see Table 1).

### 2.3. Machine Learning Algorithms

BMI was considered as both a continuous and a categorical variable. We took advantage of several algorithms, with the aim of understanding which ones work best in this specific context. In particular, we evaluated: K-nearest neighbor (KNN) [34], classification and regression tree (CART) [35], support vector machine (SVM) [36], multi-layer perceptron (MLP) [37], Ada boosting with decision tree (AB) [38], gradient boosting (GB) [39], random forest (RF) [40], and extra tree (ET) [41].

All algorithms were used for both the classification and the regression problem. For the regression analysis, we employed Lasso [42] and Elastic Net Regression [43] as additional algorithms.

We employed the Scikit-learn machine learning library in all our experiments. For all the algorithms, we used the default parameters with the only exception of the random state one, that we provided when possible to ensure reproducibility of results.

### 2.4. Approach and Evaluation Criteria

First of all, the dataset has been preprocessed to deal with missing values. Since the subjects with missing values not only had one but had many (five to seven psychological variables were missing out of a total of seven), we decided to simply remove those subjects from the dataset. In this way, the number of subjects in the dataset has gone from 320 to 221. Moreover, the dataset was divided in two different parts, one for the training phase, composed of the 80% of subjects (i.e., 176), and the other one for the testing phase, composed of the remaining 20% of subjects (i.e., 45). Then, for each one of the three steps described in Section 2.1, in the training phase, we employed the stratified k-fold cross validation, a technique used to reduce the bias deriving from random sampling [44]. We chose to use the number of folds equal to four, considering that the size of the dataset does not allow the use of the typical ten-fold cross validation. In fact, by dividing the training set into 10 folds, at each iteration, the overweight class in validation would only count 2 elements. Instead, using 4 folds, at each iteration, the overweight class in validation counts 5 elements, number which is also consistent with the size of the class in the test set. Before each training phase, the data were scaled, subtracting the average value and dividing by the standard deviation.

With regard to the classification, Table 1 shows that the dataset suffers the problem of imbalance among the three classes. In fact, the subjects of the class Obesity are more than five times the ones of the class Overweight. This imbalance could lead to predictions that are more accurate on the majority class than on a minority class, resulting in a bias in favour of the majority class. To deal with this problem, we took advantage of a resampling technique with the aim of over-sampling the minority classes. In particular, we employed the synthetic minority over-sampling technique (SMOTE) [45]. This technique exploits K-nearest neighbour in the feature space to generate synthetic examples of the minority class. In this way, during training, the number of examples for each class will be always the same.

A final consideration is due to the evaluation metrics. To assess the performance of classifiers, we employed a global metric, namely the F1-score, that is the harmonic average of the precision and recall together with two class-specific metrics, the sensitivity and specificity to measure the ability of the classifiers to predict true positives and true negatives. The prediction accuracy of our regressors has been evaluated with two different measures: mean absolute error (MAE) and Pearson correlation coefficient (PCC). The MAE measures the prediction error (i.e., the average deviation between the real BMI values and the predicted ones). The PCC quantifies the degree of the linear association between real and predicted BMI values. The reason to couple MAE and PCC is that when the values are all distributed near the average, a naive regressor that predicts always the mean value, achieve good performance. In such case, the PCC will instead be low, allowing to highlight, and consequently to avoid, such a problem.

## 3. Results

### 3.1. BMI Prediction Using Psychological Variables

**Figure 1:**
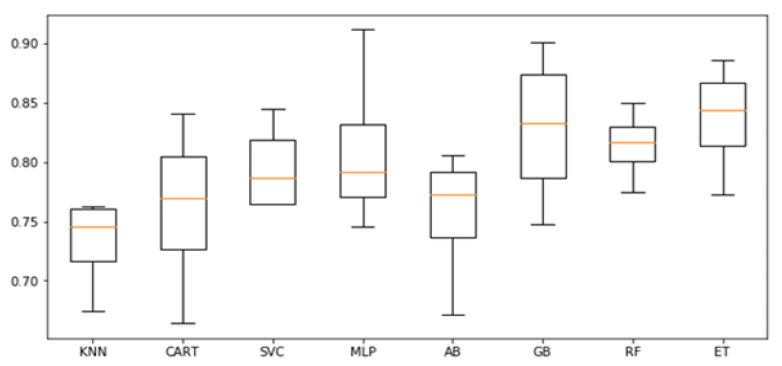
Classification: F1-scores of the cross-validation on the training set.

To answer the first research question, we investigate the employment of psychological variables to predict BMI values and classes. We conducted a first analysis employing all the algorithms described in the previous Section, taking advantage of the 4-fold cross validation. In Figure 1, we report the F1-scores obtained while predicting BMI classes with all the psychological variables available. In general, algorithms are able to predict the BMI classes. As shown, the best performances were achieved by the extra tree classifier, with an average F1-score of 0.84. However, MLP, GB, and RF were also able to achieve average F1-scores greater than 0.8. 

After this initial assessment, we conducted a tuning phase on the most promising algorithms: MLP, GB, RF, and ET. We also varied the main hyperparameters of each algorithm employing a grid search approach, hence considering all the hyper-parameters combinations. The full list of values considered for each parameter and algorithm is reported in Table 2. For each algorithm, the best combination is highlighted in bold in the Table.

After finding the hyperparameters that enable the algorithms to perform best on the training set, we have evaluated them on the test set. Table 3 reports the obtained F1-scores on the training set, always employing the 4 folds cross validation, and on the test set. As shown, on the test set, both GB and ET were able to reach F1-scores equal to 0.82. Such results highlight how it is possible to predict the BMI class using the aforementioned psychological variables with a good accuracy.

We then tackled the regression analysis. Parallel to the classification analysis, we firstly analyzed the performances of all the algorithms. Table 4 reports both the mean absolute error and the Pearson Correlation Coefficient obtained by each algorithm. As shown, the best performance was achieved by the Lasso and Elastic Net, with a MAE equal to 4.35 and the PCCs respectively of 0.81 and 0.80, indicating a strong correlation between predictions and real values. Slightly worse results were instead obtained by KNN, GB, RF, and ET.

Also in this case, we conducted a tuning phase, varying the hyperparameters of: LASSO, EN, KNN, GB, RF, and ET. The full list of values considered for each parameter and algorithm is reported in Table 5. As in the previous case, we have employed a grid search approach. For each algorithm, the best combination is highlighted in bold in the Table.

Table 6 reports the MAE and the PCC on both the training and test set. As shown, the best performance on both the training and test set is achieved by the gradient boosting with an average error of 4.14 and 5.27. For both, there is a strong correlation between the predictions and the real values, as highlighted by the PCC. Instead, the worst performances on the test set were achieved by Lasso and EN, that were the ones that initially performed better. However, even when tackling the problem as a regression one, we were able to predict BMI values starting from psychological variables.

### 3.2. Evaluation of the Impact of Positive and Negative Psychological Variables on Prediction

To answer the second research question, we contrasted the performance of the various machine learning algorithms, when trained on positive and negative psychological variables separately. We started with the classification problem. We employed the same approach of the previous Section, using the same split for the cross-validation and the same parameters for the algorithms. Table 7 reports the F1-scores obtained by the algorithms when trained with positive (Positive Variables column) and negative (Negative Variables column) psychological variables. 

As shown, there is not much difference between the performance obtained when training algorithms with all the psychological variables and the one obtained when training algorithms with only negative psychological variables. The same cannot be said of the algorithms trained with positive psychological variables. In fact, the performance falls significantly. These results highlight the fact that positive psychological variables do not influence BMI, contrary to the negative ones.

We then replicated the experiments on the regression analysis. Even in this case, we used the same split for the cross-validation and the same parameters for the algorithms. Table 8 reports both the mean absolute error and the Pearson correlation coefficient for both the algorithms trained with positive (Positive Variables column) and negative (Negative Variables column) psychological variables. The reported results confirm the ones obtained with classification algorithms. The algorithms trained with negative psychological variables present similar performances of the ones obtained by algorithms trained with all psychological variables. Thus, in many cases, the MAE of the algorithms trained on positive variables is almost two times the MAE of the algorithms trained on negative ones.

### 3.3. Evaluation of the Impact of the Single Negative Psychological Variables on Prediction

Finally, to answer the third research question, we trained our machine learning algorithms removing, in turn, each negative psychological variable: Depression (DE), Trait anxiety (TA), Binge eating (BE), and Expressive suppression (ES). In this way, we can understand which variable has more impact on the predictive capabilities of the algorithms. We first tackled the classification problem. We focused only on the training set, employing the 4-fold cross validation. Table 9 reports the F1-scores obtained by the algorithms when trained without one of the psychological variables. From the results, it is clear that the psychological variable that has most impact on the predictive capabilities of the algorithms is Depression. In fact, removing such a variable leads to a deterioration in performance of 0.2 on average (column No DE). Instead, removing any other variable does not significantly affect the performances, as shown by the values reported in columns No TA, No BE, and No ES.

We then repeated the same analysis evaluating the prediction of BMI values. Even in this case, we analysed only the training set, using the 4-fold cross validation. Table 10 reports both the mean absolute error and the Pearson correlation coefficient for the algorithm trained, in turn, without one of the negative psychological variables. The obtained results confirm the ones obtained with classification algorithms. Removing the Depression variable has a greater impact than the removal of any other variable. In fact, the performances get worse by about 2 on average.

## 4. Discussion

The current study aimed at exploring whether BMI values can be predicted from psychological parameters by using ML techniques. ML techniques represent a powerful set of algorithms that can derive useful knowledge for the medical field in general and for obesity more specifically, as they can help us to improve our understanding of such pathology and our capacity to predict it with greater precision [46]. Risk prediction of adverse health conditions and events is a primary goal of much health research, and this study had the objective to provide evidence about the role of psychological factors as either risk (negative affectivity) or protective (positive affectivity) determinants of BMI levels through non-conventional statistical techniques.

Several ML algorithms were used to test theoretical models about the relationship between psychological variables and BMI. First of all, we can highlight how regardless of how the BMI is conceptualized (i.e., as a continuous value or as a categorical variable), the results are the same, without particular differences. For this reason, in the presentation of the answers to the research questions, we will not differentiate between the two types of problems. From the results presented in Section 3.1, it is clear that the answer to the first research question is affirmative. In fact, using affect-related variables it is possible to predict the BMI with a good level of accuracy. To answer the second research question, instead, we have used as input positive and negative affect-related variables separately. The results reported in Section 3.2 showed that BMI can be better predicted by the set of negative affect-related variables, such as depression, anxiety, and emotion suppression, whereas variables with more positive contents, such as happiness and emotion regulation, did not seem to play a predictive role over BMI. Hence, in the third step of our experiments, we considered only negative affect-related variables, leaving out one variable in turn to respond to the last research question. Among the psychological variables that we considered, depression seemed to have the strongest predictive power. In fact, the results presented in Section 3.3 it is clear how the removal of depression generally leads to a significant lowering of the predictive capabilities of the machine learning algorithms, which does not happen for the other variables. Such a finding reinforces already published results that have highlighted the role of depression [22]. These results add to the literature on ML and obesity by focusing on relevant psychological parameters for the prediction of BMI, and suggest that affective variables, particularly depression, should be considered in preventive and treatment care of BMI-related problems, especially in the case of elevated BMI and obesity. 

To our knowledge, no prior investigation has used ML techniques to test for the predictive effects of emotional and affective variables over BMI values. In fact, already published studies where ML was employed took into account physiological parameters such as voice signals [15] and face images [16,17]. However, further research should combine these findings by taking into account both medical and psychological parameters simultaneously. This would help to verify and compare the predictive role of these variables.

We must address the limitations of the current study. Firstly, it did not employ newly collected data, thus making our inferences limited. However, it allowed us to have a basis for comparison and to test for the reproducibility of previous findings [23,24,25,26]. Secondly, this study suffers from a number of methodological flaws, such as cross-sectional study design and a prevalence of self-evaluation (with the exclusion of BMI values which were directly assessed by the medical staff), as already discussed in [22]. Those issues should be solved in future studies. Thirdly, from a technical perspective, the main limitation of this work is surely the restricted number of subjects. Increasing the size of the dataset, possibly in a balanced way, would help to strengthen the obtained results. Moreover, it would also allow the use of more powerful, yet data-hungry, algorithms, such as deep neural networks. Lastly, aside from BMI, the present study took into account psychological and demographic variables only. However, given the multifactorial nature of weight-related disorders, future studies need to include relevant medical and ‘lifestyle’ variables which may contribute to the explanation of present results (e.g., actual calories intake, weekly exercise, social support).

In conclusion, despite these limitations, present findings provide statistically strong information regarding the possibility to predict BMI values by means of a set of psychological variables with negative contents. Particularly, this is one of the first studies investigating the predictive role of psychological factors over a condition such as obesity, through ML algorithms [47]. These data highlight the importance of considering the affective component of individual’s experience for a better and more complete understanding of weight-related disorders, as it can inform psychological interventions and treatment approaches, as well as improve preventive and therapeutic strategies. Yet, the use of ML has several advantages, as it outperforms traditional statistics, can be used to compare the impact of more variables on the prediction of the chosen outcome, and can handle any kind of variable. However, in order to improve the strength of these findings, future research aimed at overcoming present study limitations is required.

## Figures and Tables

**Table 1:** Number of subjects for each BMI class.

BMI Class	Number of Subjects
Normal Weight	60
Overweight	25
Obesity	136

**Table 2:** Hyper-parameters and values tested during tuning for the classification.

Algorithm	Parameter	Values
MLP	Activation Function	identity, logistic, tanh, **relu**
	Solver	lbfgs, **sgd**, adam
	Max Iterations	200, **500**, 1000
	Alpha	**0.1**, 0.01, 0.001, 0.0001
	Hidden layer size	**50**, 100, 150, 200
RF	Min Samples Leaf	1, 3, **5**
	Min Samples Split	**2**, 4, 6
	Max Depth	3, **5**, 8
	Max Features	**log2**, sqrt
	Criterion	gini, **entropy**
	Bootstrap	**true**, false
	Number of Estimators	50, 100, **200**, 500
GB	Learning Rate	0.01, 0.05. 0.1, **0.2**
	Min Samples Leaf	1, 3, **5**
	Min Samples Split	**2**, 4, 6
	Max Depth	3, 5, **8**
	Max Features	**log2**, sqrt
	Criterion	**friedman mse**, mae
	Subsample	0.5, **0.75**, 1
	Number of Estimators	50, 100, **200**, 500
ET	Min Samples Leaf	1, **3**, 5
	Min Samples Split	2, **4**, 6
	Max Depth	3, 5, **8**
	Max Features	**log2**, sqrt
	Criterion	gini, **entropy**
	Number of Estimators	50, 100, 200, **500**

**Table 3:** Classification: F1-score, specificity/sensitivity on the test set, after tuning.

Algorithm	Class	Sensitivity	Specificity	F1-Score
MLP	Normal Weight	0.67	0.91	0.81
	Overweight	0.60	0.90	
	Obesity	0.89	0.88	
GB	Normal Weight	0.83	0.91	0.85
	Overweight	0.60	0.93	
	Obesity	0.89	0.94	
RF	Normal Weight	0.92	0.91	0.89
	Overweight	0.60	0.97	
	Obesity	0.93	0.94	
ET	Normal Weight	0.75	0.88	0.82
	Overweight	0.60	0.97	
	Obesity	0.89	0.82	

**Table 4:** Regression: Mean Absolute Error and Pearson Correlation Coefficient of the cross-validation on the training set.

Algorithm	MAE	PCC
LASSO	4.35	0.81
EN	4.35	0.8
CART	5.93	0.63
KNN	4.37	0.79
SVR	5.33	0.75
MLP	9.44	0.5
AB	4.62	0.76
GB	4.58	0.76
RF	4.65	0.77

**Table 5:** Hyper-parameters and values tested during tuning for the regression.

Algorithm	Parameter	Values
LASSO	Alpha	**1.0**, 0.75, 0.5, 0.25
EN	Alpha	**1.0**, 0.75, 0.5, 0.25
KNN	N Neighbors	3, 7, 11, 15, **21**
	Leaf Size	**1**, 2, 3, 5
	Weights	uniform, **distance**
	Algorithm	auto, ball tree, kd tree, **brute**
RF	Min Samples Leaf	1, **3**, 5
	Min Samples Split	**2**, 4, 6
	Max Depth	3, **5**, 8
	Max Features	**log2**, sqrt
	Criterion	mse,**mae**
	Bootstrap	**true**, false
	Number of Estimators	50, **100**, 200, 500
Gb	Learning Rate	0.01, **0.05**, 0.1, 0.2
	Min Samples Leaf	1, 3, **5**
	Min Samples Split	**2**, 4, 6
	Max Depth	3, 5, **8**
	Max Features	**log2**, sqrt
	Criterion	friedman mse, **mae**
	Subsample	0.5, **0.75**, 1
	Number of Estimators	50, **100**, 200, 500
ET	Min Samples Leaf	**1**, 3, 5
	Min Samples Split	**2**, 4, 6
	Max Depth	3, 5, **8**
	Max Features	**log2**, sqrt
	Criterion	mse,**mae**
	Number of Estimators	50, **100**, 200, 500

**Table 6:** Regression: Mean Absolute Error and Pearson Correlation Coefficient on the training and test set, after the tuning phase.

Algorithm	4-Fold CV		Test	
	MAE	PCC	MAE	PCC
LASSO	4.35	0.81	6.00	0.72
EN	4.35	0.80	6.52	0.70
KNN	4.31	0.76	5.50	0.76
GB	4.14	0.79	5.27	0.75
RF	4.26	0.79	5.31	0.78
ET	4.41	0.78	5.57	0.76

**Table 7:** Classification: positive vs. negative variables, F1-scores of the cross-validation on the training set.

Algorithm	Class	Positive Variables			Negative Variables		
		Sen	Spec	F1	Sen	Spec	F1
KNN	Normal Weight	0.38	0.66	0.43	**0.67**	**0.90**	**0.79**
	Overweight	0.35	0.69		**0.45**	**0.83**	
	Obesity	0.41	0.78		**0.86**	**0.96**	
CART	Normal Weight	0.33	0.67	0.44	**0.67**	**0.89**	**0.77**
	Overweight	0.15	0.80		**0.30**	**0.85**	
	Obesity	0.50	0.74		**0.87**	**0.91**	
SVC	Normal Weight	0.48	0.73	0.51	**0.79**	**0.91**	**0.86**
	Overweight	0.30	0.74		**0.45**	**0.93**	
	Obesity	0.51	0.74		**0.96**	**0.96**	
MLP	Normal Weight	0.50	0.69	0.52	**0.71**	**0.90**	**0.82**
	Overweight	0.25	0.78		**0.40**	**0.90**	
	Obesity	0.53	0.77		**0.94**	**0.94**	
AB	Normal Weight	0.42	0.68	0.49	**0.69**	**0.87**	**0.70**
	Overweight	0.15	0.83		**0.55**	**0.76**	
	Obesity	0.56	0.63		**0.68**	**0.94**	
GB	Normal Weight	0.40	0.75	0.51	**0.69**	**0.91**	**0.81**
	Overweight	0.25	0.81		**0.35**	**0.92**	
	Obesity	0.57	0.59		**0.96**	**0.88**	
RF	Normal Weight	0.38	0.72	0.47	**0.75**	**0.89**	**0.82**
	Overweight	0.20	0.80		**0.35**	**0.92**	
	Obesity	0.52	0.56		**0.94**	**0.93**	
ET	Normal Weight	0.38	0.73	0.49	**0.75**	**0.88**	**0.81**
	Overweight	0.20	0.78		**0.30**	**0.91**	
	Obesity	0.55	0.62		**0.93**	**0.93**	

**Table 8:** Regression: positive vs. negative variables, Mean Absolute Error and Pearson Correlation Coefficient of the cross-validation on the train set.

Algorithm	Positive Variables		Negative Variables	
	MAE	PCC	MAE	PCC
LASSO	8.05	0.16	**4.41**	**0.83**
EN	8.03	0.16	**4.4**	**0.83**
CART	9.96	0.23	**6.12**	**0.69**
KNN	8.47	0.1	**4.37**	**0.8**
SVR	8.19	0.15	**4.69**	**0.81**
MLP	9.88	0.08	**7.74**	**0.62**
AB	8.01	0.17	**4.35**	**0.82**
GB	8.04	0.28	**4.34**	**0.82**
RF	8.13	0.3	**4.34**	**0.83**
ET	8.14	0.33	**4.18**	**0.84**

**Table 9:** Classification: F1-scores of the cross-validation on the training set, removing in turn one negative psychological variable.

Algorithm	Class	No DE			No TA			No BE			No ES		
		Sen	Spec	F1	Sen	Spec	F1	Sen	Spec	F1	Sen	Spec	F1
KNN	N.W.	0.60	0.77	**0.59**	0.63	0.91	0.80	0.63	0.85	0.74	0.56	0.92	0.78
	Over.	0.30	0.76		0.55	0.84		0.20	0.83		0.45	0.82	
	Obes.	0.58	0.82		0.89	0.96		0.86	0.94		0.92	0.96	
CART	N.W.	0.46	0.80	**0.56**	0.65	0.88	0.75	0.56	0.91	0.75	0.75	0.88	0.78
	Over.	0.25	0.83		0.30	0.86		0.35	0.85		0.30	0.90	
	Obes.	0.66	0.62		0.86	0.87		0.90	0.85		0.88	0.88	
SVC	N.W.	0.65	0.81	**0.63**	0.75	0.92	0.85	0.54	0.88	0.77	0.73	0.91	0.84
	Over.	0.35	0.80		0.50	0.91		0.35	0.85		0.45	0.91	
	Obes.	0.63	0.79		0.95	0.96		0.94	0.97		0.95	0.94	
MLP	N.W.	0.65	0.78	**0.64**	0.69	0.91	0.82	0.75	0.88	0.81	0.69	0.91	0.82
	Over.	0.30	0.87		0.50	0.89		0.30	0.90		0.45	0.89	
	Obes.	0.68	0.75		0.93	0.94		0.93	0.96		0.94	0.94	
AB	N.W.	0.52	0.81	**0.57**	0.77	0.87	0.75	0.73	0.88	0.72	0.75	0.85	0.76
	Over.	0.30	0.80		0.30	0.83		0.30	0.78		0.35	0.85	
	Obes.	0.61	0.66		0.79	0.93		0.75	0.96		0.80	0.94	
GB	N.W.	0.54	0.83	**0.62**	0.67	0.89	0.79	0.67	0.88	0.79	0.75	0.91	0.82
	Over.	0.25	0.86		0.30	0.90		0.30	0.90		0.40	0.90	
	Obes.	0.70	0.63		0.94	0.90		0.94	0.90		0.93	0.94	
RF	N.W.	0.54	0.80	**0.61**	0.69	0.88	0.79	0.65	0.89	0.79	0.79	0.89	0.82
	Over.	0.25	0.85		0.30	0.90		0.35	0.90		0.35	0.92	
	Obes.	0.69	0.68		0.92	0.90		0.94	0.90		0.93	0.93	
ET	N.W.	0.58	0.81	**0.62**	0.71	0.90	0.81	0.60	0.88	0.78	0.81	0.91	0.83
	Over.	0.20	0.85		0.35	0.91		0.35	0.90		0.35	0.92	
	Obes.	0.69	0.69		0.94	0.90		0.94	0.90		0.94	0.93	

**Table 10:** Regression: Mean Absolute Error and Pearson Correlation Coefficient of the cross-validation on the training set, removing in turn one negative psychological variable.

Algorithm	No DE		No TA		No BE		No ES	
	MAE	PCC	MAE	PCC	MAE	PCC	MAE	PCC
LASSO	6.83	0.58	5.01	0.78	4.59	0.82	4.47	0.82
EN	6.83	0.58	5	0.78	4.58	0.82	4.46	0.82
CART	9.07	0.3	5.92	0.69	5.8	0.72	6.39	0.67
KNN	6.68	0.57	4.86	0.78	4.41	0.83	4.6	0.8
SVR	6.92	0.56	4.86	0.79	4.82	0.81	4.46	0.81
MLP	8.18	0.44	7.26	0.61	7.09	0.66	8.02	0.62
AB	7.25	0.54	4.58	0.8	4.56	0.82	4.63	0.8
GB	6.69	0.55	4.6	0.79	4.41	0.82	4.66	0.79
RF	6.77	0.53	4.54	0.81	4.54	0.82	4.75	0.79
ET	7.09	0.51	4.45	0.82	4.55	0.83	4.67	0.8

## Data Availability

Data sharing not applicable.
